# High consumption of ultra-processed food may double the risk of subclinical coronary atherosclerosis: the Aragon Workers’ Health Study (AWHS)

**DOI:** 10.1186/s12916-020-01678-8

**Published:** 2020-08-13

**Authors:** Henry Montero-Salazar, Carolina Donat-Vargas, Belén Moreno-Franco, Helena Sandoval-Insausti, Fernando Civeira, Martín Laclaustra, Pilar Guallar-Castillón

**Affiliations:** 1 Department of Preventive Medicine and Public Health, School of Medicine, Universidad Autónoma de Madrid, CEI UAM+CSIC, Avda. Arzobispo Morcillo, n 4, 28029 Madrid, Spain; 2 Unit of Nutritional and Cardiovascular Epidemiology, Environmental Medicine Institute (IMM), Karolinska Institutet, Stockholm, Sweden; 3 CIBERESP (CIBER of Epidemiology and Public Health) Instituto de Salud Carlos III, Madrid, Spain; 4 Instituto de Investigación IdiPaz, Madrid, Spain; 5 IMDEA-Food Institute CEI UAM+CSIC, Madrid, Spain; 6 IIS Aragón, Hospital Universitario Miguel Servet, Universidad de Zaragoza, Zaragoza, Spain; 7 CIBERCV Instituto de Salud Carlos III, Madrid, Spain; 8 Department of Microbiology, Preventive Medicine and Public Health, University of Zaragoza, Zaragoza, Spain; 9 Department of Nutrition, Harvard T.H. Chan School of Public Health, Boston, MA USA; 10 Agencia Aragonesa para la Investigación y el Desarrollo (ARAID), Zaragoza, Spain

**Keywords:** Ultra-processed food, Subclinical coronary atherosclerosis, Coronary calcium, Cross-sectional cohort study, Nutritional epidemiology

## Abstract

**Background:**

Ultra-processed food (UPF) consumption, which is increasing worldwide, has recently been associated with an increased risk of death and cardiovascular disease. We aimed to assess whether consumption of UPF is directly associated with subclinical coronary atherosclerosis in middle-aged men.

**Methods:**

A computed tomography scan was performed on 1876 men from the Aragon Workers’ Health Study, recruited from January 2011 to December 2014, to assess coronary calcium. All participants were free of coronary heart disease. Dietary intake was collected by a validated 136-item semi-quantitative food frequency questionnaire. UPF was defined according to the NOVA classification. Associations between consumption of total energy-adjusted UPF and Coronary Calcium Agatston Score (CACS)—categorized into CACS of 0, > 0 and < 100, and ≥ 100—were cross-sectionally assessed by generalized ordered logistic regression adjusted for main confounders.

**Results:**

No coronary calcium was detected in 60.2% of the participants, whereas 10.2% had a CACS ≥ 100. A significant dose-response association was observed between energy-adjusted UPF consumption and the risk of having a CACS ≥ 100, when compared with those in the lowest CACS categories (CACS of 0 together with CACS > 0 and < 100). The fully adjusted ORs (95% CI) of having a CACS ≥ 100 across quartiles of energy-adjusted UPF consumption (approximately 100 g/day in the lowest quartile (ref.) and 500 g/day in the highest) were 1.00 (ref.), 1.50 (0.93, 2.42), 1.56 (0.96, 2.52), and 2.00 (1.26, 3.16), *p* trend .005.

**Conclusion:**

In this middle-aged worker’s sample, approximately 500 g/day of UPF consumption was associated with a 2-fold greater prevalence of subclinical coronary atherosclerosis than consuming only 100 g/day, independently of total energy intake and other well-established cardiovascular risk factors.

## Background

The food and beverage industry has experienced high growth in recent years, and the consumption of ultra-processed food (UPF) has substantially increased, fostered by attractive packaging and intensive marketing [1]. UPF consumption in Spain is low to moderate [2], but it is increasing rapidly. While in 1990, UPF consumption represented 11% of daily energy intake in Spain, it has almost tripled in 10 years [3]. In 2000, the mean contribution of UPF in total energy intake was about 35% in Spain and Italy, but it reached up to 60% in the Netherlands, Sweden, Norway, Denmark, and the UK general population [4].

UPF is formulated mostly or entirely from substances derived from food together with additives, with little, if any, intact food. Processing entails greater durability, tastier flavors, and readiness to consume at a very low price. UPF is characterized by poor nutritional value and high energy density with low fiber and micronutrient content, as well as high amounts of sodium, saturated and trans fats, and simple sugars [5, 6]. On the one hand, all aforementioned detrimental nutrients have been individually associated with subclinical atherosclerosis and cardiovascular disease (CVD) [7–9]. On the other hand, UPF also contains a great diversity of additives, many of which have shown adverse effects on the vascular system in experimental studies [10–12]. In particular, the additive phosphate, present in almost all UPF, is involved in atherosclerosis by inducing vascular calcification both in vitro and in vivo [13]. Finally, UPF consumption replaces the intake of other unprocessed or minimally processed food and freshly prepared meals that have beneficial nutritional attributes, also affecting health in an indirect way.

Recently, in the large prospective NutriNet-Santé cohort, an absolute increment of 10% of UPF in the diet has been associated with a 12%, 13%, and 11% statistically significant increase in the rates of overall CVD, coronary heart disease (CHD), and cerebrovascular disease, respectively [14]. Likewise, consumption of UPF has been associated with cardiometabolic conditions [15], such as overweight or obesity [16], hypertension [17], dyslipidemia [18], and diabetes [19], as well as with CVD mortality or total mortality [2, 20, 21].

Atherosclerosis, which underlies CVD, is a complex disease in which fat, inflammation cells, scar tissue, and deposits of calcium accumulate within the walls of the arteries [22]. The presence of calcium in the coronary arteries is an indicator of subclinical atherosclerotic disease and a marker of coronary damage [23], as well as a strong and independent predictor of future coronary heart disease [24, 25]. Current guidelines endorse the measurement of coronary calcium to improve risk prediction of coronary disease in selected asymptomatic individuals [26, 27]. Accordingly, high calcium in coronary arteries has been consistently associated with general coronary disease [28], myocardial infarction [28, 29], heart failure [29], and stroke [30].

To date, no epidemiological study has yet evaluated the direct impact of UPF consumption on the coronary arteries in asymptomatic subjects. The aim of this study is therefore to examine the association between UPF consumption and subclinical coronary atherosclerosis in a sample of middle-aged subjects with a low prevalence of clinical comorbidities.

## Methods

### Study design and population

**Fig. 1:**
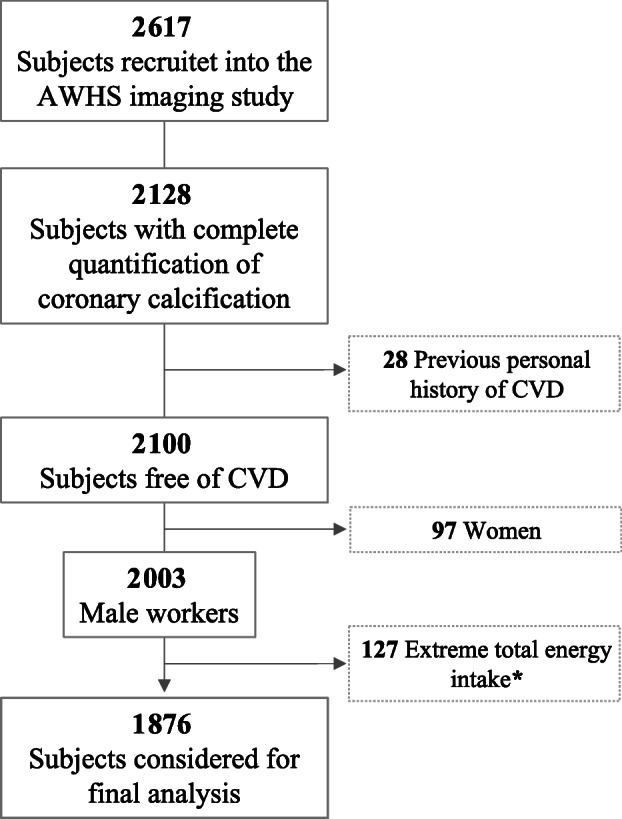
Flow chart for the study association: Coronary Artery Calcium Score and ultra-processed food consumption. *Total energy intake of < 600 or > 4200 kcal in men was considered extreme values. Subjects recruited in the imaging AWHS. AWHS, Aragon Workers’ Health Study; CVD, cardiovascular disease

The present cross-sectional study includes a sample of participants from the Aragon Workers’ Health Study (AWHS), the design of which has been described in detail elsewhere [31, 32]. Study participants are workers of Opel Spain automobile assembly plant located in Figueruelas (Zaragoza, Spain) that were recruited during a standardized clinical exam in 2009–2010 (participation rate 95.6%). In addition, between January 2011 and December 2014, all participants who were aged 40–60 years old (34% of initial participants) were invited to undergo a coronary calcification scan and provided blood and urine samples for the study biobank, as well as to answer a comprehensive questionnaire on cardiovascular and lifestyle factors, including diet. No relevant differences were detected in baseline characteristics between total participants and those who undergo the coronary calcification scan. Among the 2617 workers (all Caucasian) recruited into the AWHS imaging study, 2128 had complete quantification of coronary calcification. Likewise, those subjects with previous history of CVD (*n* = 28), women (*n* = 97), and those with an extreme total energy intake (< 600 or > 4200 kcal) (*n* = 127) were excluded, resulting in a final sample of 1876 participants (Fig. 1).

### Data collection

Demographic information including age, sex, marital status, educational level, smoking, sleep duration (both on weekdays and weekends), and diabetic status was obtained by questionnaires. Also, leisure-time physical activity and time spent in sedentary activities were assessed using a formerly validated questionnaire, i.e., the Health Professionals’ Follow-up physical activity questionnaire [33], that was highly correlated with objective measurements using triaxial accelerometer (RT3 Triaxial Research Tracker) as reference (Spearman’s correlation coefficient of 0.51; *p* < 0.001). Participants were asked about the time devoted to 17 different activities during the preceding year, and leisure-time physical activity was expressed in metabolic equivalents (METs)-h/week.

Serum samples were obtained, and cholesterol, triglycerides, and glycemia were measured. Study participants went through a standardized clinical exam with blood pressure (BP) and heart rate measurements. BP was measured three consecutive times using an automatic oscillometric sphygmomanometer after being seated for 5 min. Anthropometrics, including height, weight, and waist circumference, were also measured following standardized procedures, and body mass index (BMI, in kg/m^2^) was calculated.

Physicians and nurses collecting these data underwent specific training and standardization programs organized by the study investigators. Compliance with study procedures was routinely monitored, and deviations were corrected. The study conforms to the ISO9001-2008 quality standard.

### Dietary assessment and ultra-processed food consumption

Their usual diet over the preceding year was assessed using a 136-item semi-quantitative food frequency questionnaire (FFQ) previously validated and repeatedly re-evaluated in Spain [34–36]. This FFQ considers seasonal variations and differences in food consumption between weekdays and weekend patterns. We measured the frequency of food consumption in nine categories (ranging from never or almost never to more than six servings per day), also including a standard portion size for each food item. To estimate daily consumption for each food item, we multiplied the portion size by the frequency of consumption.

We listed all food and beverage items of the FFQ according to NOVA classification [6] which organizes food into four groups based on the scope and purpose of industrial processing. The first group includes unprocessed or minimally processed food, which is fresh or modified by filtering, freezing, drying, or pasteurization, with no addition of salt, sugar, oils, or fats. The second group contains processed culinary ingredients. These are substances derived from nature, but have undergone processes such as pressing, refining, or milling and might contain additives to preserve the original properties (i.e., salt, sugar, honey, vegetable oils, butter, lard, and vinegar). The third group comprises processed food. This is food which has undergone preservation or preparation methods (e.g., smoking, curing, or fermentation) in order to last longer or to enhance their sensory qualities. Examples include canned or bottled vegetables and legumes, fruit in syrup, canned fish, cheese, freshly made bread, and salted/sugared nuts and seeds. The fourth group comprises ultra-processed food and drink products that are made predominantly or entirely from industrial substances and contain little or no whole food. Under this classification, we can find products such as hamburgers, frozen pizza and pasta dishes, French fries, breads, cakes, industrially manufactured biscuits (cookies), jams and confectionery, margarines, cereal bars, soft drinks and other sugary beverages such as sugared milk and fruit drinks, fruit yogurts, instant packaged soups and noodles, and sweet or savory snacks.

For each participant, total UPF consumption in grams per day (g/day) was calculated summing up the consumption from each UPF item included in the fourth group of the NOVA classification (Additional File 1: Table S1). The grams per day of UPF were adjusted for total energy intake by the residual method [37].

### Outcome assessment: Coronary Agatston Calcium Score

Coronary calcium quantification was performed using non-contrast ECG-gated prospective acquisition by a 16-multidetector computed tomography scanner (Philips). Agatston’s method is a summed score of all coronary calcified lesions, accounting for both the total area and the maximum density of coronary calcium. A high Coronary Calcium Agatston Score (CACS) is a strong indicator of extensive disease with a significant amount of calcium deposits. CACS remains the reference standard and the most commonly used coronary artery calcium score in clinical practice [38].

CACS was divided into three consecutive categories: 0, > 0 and < 100, and ≥ 100. Having a CACS > 0 represents the presence of calcium, and surpassing a threshold of ≥ 100 is considered as having a moderate to severe subclinical coronary atherosclerosis and has been associated with increases in coronary heart disease rates [39].

### Statistical analysis

Participants were categorized into quartiles of daily grams of UPF consumption after adjusting for total energy intake using the residual method [37]. The analyses were also carried out using quartiles of daily percentage of energy derived from UPF, revealing very similar results (data not presented).

The adequacy of the proportional odds assumption across response categories (a requirement for conducting ordered logistic regression) was examined by using tests for proportionality (Wald’s test and Brant’s test). We found a violation of this assumption (*p* values < 0.05). Accordingly, we estimated the odds ratio (OR) and corresponding 95% confidence intervals (CI) for having progressed to categories of more coronary artery calcium using generalized ordered logistic models (gologit/partial proportional odds model). These models are less restrictive than the proportional-odds/parallel-lines models and do not assume the equality of slopes among categories. This approach yields two ORs, one describing the relationship between the lowest vs. the two highest categories of the response variable (CACS), and the other describing the relationship between the two lowest categories vs. the highest one [40].

We additionally performed a standard binary logistic regression for the collapsed categories to estimate the OR for CACS > 0 (compared with CACS of 0) and for CACS ≥ 100 (compared with CACS < 100), to provide estimations which can easily be understood as they are widely used. So, we provide two estimators to describe the association between UPF consumption and CACS. To calculate the *p* for linear trend, the mean concentrations of UPF in each quartile were used and treated as a continuous variable in the model. Likewise, the standard binary logistic regression was used to perform restricted cubic splines with 3 knots (at the 10th, 50th, and 90th percentiles of the distribution) to visualize flexible dose-response associations.

We used three models with progressive adjustment for covariates that can operate as confounders [41]. Model 1 was adjusted for age (continuous, years); model 2 was further adjusted for demographic and lifestyle factors: marital status (married, not married), education (middle school, high school, professional training, and college), smoking status (never, former, and current smoker), physical activity (in MET-h/week), time spent sleeping during the weekdays (number of hours of sleep, continuous), time spent sleeping during the weekend (number of hours of sleep, continuous), and dietary factors such as alcohol consumption (g/day), total fiber (g/day), cholesterol intake,(mg/day), and total energy intake (kcal); and model 3 was further adjusted for cardiometabolic risk factors: total cholesterol in blood (mg/dL), HDL cholesterol in blood (mg/dL), systolic and diastolic blood pressure (mmHg), BMI (< 25, 25 to < 30, ≥ 30 kg/m^2^), and diabetes (yes, no).

To maximize the use of available information, missing values on education (< 1%) and smoking (< 1%) were included in a separate category. Missing values on hours of sleep during the week (< 1%) and BMI (< 1%) were imputed by predicted values from a multivariable regression model containing the corresponding explanatory variables. Missing values on diabetes (3%) were considered as having a non-disease status.

We tested for interactions between UPF consumption and age, BMI, smoking status, alcohol consumption, and physical activity, on the multiplicative scale using the likelihood ratio test, comparing binary logistic models with and without an interaction term.

Analyses were performed with Stata/SE, version 15.1 (StataCorp, College Station). Statistical significance was set at the two-sided 0.05 level.

## Results

All participants were Caucasian males with a mean age of 51 ± 3.7 years. The average UPF consumption ranged from 117 ± 56 g/day in the lowest quartile to 484 ± 217 g/day in the highest one. Those in the highest quartile of UPF consumption were less frequently current smokers; performed less physical activity; had a lower consumption of alcohol, fiber, micronutrients (vitamins and minerals); and had lower HDL cholesterol levels. Likewise, although non-statistically significant, those consuming more UPF were more frequently obese than those consuming less. In general, no other meaningful differences in demographic and lifestyle variables were observed across UPF quartiles (Table 1).

**Table 1:** Characteristics of the study participants according to quartiles of energy-adjusted ultra-processed food (UPF) consumption, the AWHS cohort study (*N* = 1876)

	Energy-adjusted UPF consumption (g/day)*				
	Q1 (*n* = 469)	Q2 (*n* = 469)	Q3 (*n* = 469)	Q4 (*n* = 469)	*p* value
Energy-adjusted UPF consumption (g/day)*	117 ± 56	169 ± 66	263 ± 76	484 ± 217	< .01
Total energy intake (kcal)	2988 ± 564	2685 ± 616	2793 ± 638.4	2840 ± 601	< .01
Age (years)	51.5 ± 3.6	51.3 ± 3.7	51.0 ± 3.7	50.7 ± 3.9	< .01
Married (%)	85.6 (406)	85.5 (401)	84.9 (398)	85.3 (400)	.90
Education (%)					.68
Middle school	52.6 (245)	49.1 (229)	52.2 (242)	55.0 (255)	
High school	12.0 (56)	11.8 (55)	10.1 (47)	10.1 (47)	
Professional training	31.8 (148)	33.5 (156)	34.0 (158)	30.6 (142)	
College	3.6 (17)	5.6 (26)	3.7 (17)	4.3 (20)	
Smoking (%)					< .01
Never	20.6 (96)	19.7 (92)	26.3 (123)	25.5 (119)	
Former	29.2 (136)	29.4 (137)	34.1 (159)	36.2 (169)	
Current	50.2 (234)	50.9 (237)	39.6 (185)	38.3 (179)	
Physical activity (MET-h/week)	36.22 ± 23.8	31.84 ± 21.0	31.0 ± 22.1	31.5 ± 22.5	< .01
Sleep duration (hours)					
During the week	6.3 ± 1.0	6.3 ± 0.9	6.3 ± 1.0	6.3 ± 1.0	.66
During the weekend	7.3 ± 1.1	7.3 ± 1.2	7.3 ± 1.2	7.3 ± 1.2	.92
Body mass index (%)					.35
< 25 kg/m^2^	20.5(96)	17.5 (82)	18.8 (88)	19.6 (92)	
25 to < 30 kg/m^2^	59.9 (281)	58.6 (275)	57.1 (268)	54.6 (256)	
≥ 30 kg/m^2^	19.6 (92)	23.9 (112)	24.1 (113)	25.8 (121)	
Cholesterol intake (mg/day)	456.7 ± 139.3	440.5 ± 129.4	461.6 ± 140.6	460.4 ± 140.4	.07
Total cholesterol in blood (mg/dL)	223.3 ± 36.1	222.1 ± 35.7	223.3 ± 35.4	220.0 ± 37.6	.46
HDL cholesterol in blood (mg/dL)	54.1 ± 11.9	52.4 ± 10.9	52.7 ± 11.1	52.0 ± 11.2	.03
Blood pressure (mmHg)					
Systolic	126.3 ± 14.3	125.3 ± 13.9	125.6 ± 14.8	126.6 ± 14.1	.47
Diastolic	83.2 ± 9.6	82.9 ± 9.5	82.9 ± 9.6	83.8 ± 9.1	.42
Prevalent diabetes (%)	4.5 (21)	3.4 (16)	4.3 (20)	3.8 (18)	.84
Alcohol consumption (g/day)	25.2 ± 22.2	18.8 ± 17.7	20.1 ± 20.5	20.6 ± 18.7	< .01
Total fiber* (g/day)	27.2 ± 7.7	25.2 ± 7.9	24.3 ± 7.0	23.2 ± 6.7	< .01
Omega 3 non-marine source* (g/day)	1.6 ± 0.7	1.6 ± 0.6	1.6 ± 0.5	1.5 ± 0.6	0.08
Omega 3 marine source* (g/day)	0.73 ± 0.4	0.71 ± 0.4	0.67 ± 0.4	0.63 ± 0.4	< .01
Vitamin C* (mg/day)	180.9 ± 72.8	184.4 ± 68.9	177.9 ± 61.5	178.4 ± 71.3	.45
Vitamin D* (μg/day)	5.4 ± 3.3	5.5 ± 3.0	5.2 ± 2.8	5.0 ± 2.7	.02
Vitamin A* (μg/day)	1226.4 ± 673	1220.6 ± 554	1272.4 ± 643	1206.7 ± 682	< .01
Vitamin E* (mg/day)	10.5 ± 3.6	10.9 ± 3.2	11.4 ± 3.8	11.5 ± 3.7	< .01
Vitamin B6* (mg/day)	2.5 ± 0.5	2.5 ± 0.5	2.4 ± 0.4	2.3 ± 0.5	< .01
Vitamin B9* (mg/day)	379.7 ± 72.6	369.9 ± 71.0	357.4 ± 61.6	345.3 ± 66.6	< .01
Vitamin B12* (mg/day)	10.1 ± 4.4	9.7 ± 3.4	9.8 ± 4.1	9.2 ± 4.0	< .01
Magnesium* (mg/day)	429.6 ± 69.6	426.7 ± 68.3	415.3 ± 60.6	407.3 ± 61.7	< .01
Calcium* (mg/day)	1018.9 ± 323.2	1035.8 ± 285.1	1019.0 ± 280.3	1019.9 ± 304.0	.78
Zinc* (mg/day)	15.5 ± 2.1	14.9 ± 2.0	14.5 ± 1.9	14.3 ± 1.9	< .01
Iodine* (μg/day)	301.3 ± 195.6	294.7 ± 151.1	276.2 ± 147.7	258.9 ± 156.3	< .01
Selenium* (μg/day)	132.7 ± 27.5	123.0 ± 25.8	116. 8 ± 25.7	113.1 ± 26.2	< .01

Continuous variables are presented as mean ± standard deviation and categorical variables as percentage (frequency, *n*)

*p* value estimates are based on one-way ANOVA (Bonferroni’s multiple-comparison test) for variables expressed as mean (standard deviation) or Pearson’s *χ*^2^ test for variables expressed as percentages

*UPF* ultra-processed food, *Q* quartiles

*Energy adjusted by the residual method

Regarding CACS, 60.2% of the participants (*n* = 1129) had no coronary artery calcium, 29.6% had a CACS > 0 but < 100 (*n* = 556), and the remaining 10.2% (*n* = 191) had a CACS ≥ 100 (those with a well-defined and established disease). A significant dose-response association was observed between daily consumption of UPF and the risk of progress from the two lowest categories (CACS of 0 together with CACS > 0 and < 100) to the highest category (CACS ≥ 100). The fully adjusted ORs (95% CI) of having a CACS ≥ 100 across quartiles of UPF consumption (1st quartile as reference) were 1.50 (0.93, 2.42), 1.56 (0.96, 2.52), and 2.00 (1.26, 3.16), *p* trend .005. However, there was no association between consumption of UPF and the risk of progressing from no coronary artery calcium (CACS of 0) to the two highest CACS categories (CACS > 0 and < 100 together with CACS ≥ 100) (Table 2).

**Table 2:** Progression to higher coronary artery calcium score (CACS) categories according to quartiles of ultra-processed food (UPF) consumption, using generalized ordered logistic models, the AWHS cohort study (*N* = 1876)

CACS categories	Quartiles of energy-adjusted UPF consumption (g/day)*, odds ratio (95% confidence intervals)				
	Q1	Q2	Q3	Q4	*p* trend
From the lowest category to the two highest^†^					
Model 1, OR (95% CI)	1 (ref.)	1.10 (0.84, 1.44)	1.18 (0.90, 1.55)	1.15 (0.88, 1.51)	.315
Model 2, OR (95% CI)	1 (ref.)	1.09 (0.83, 1.44)	1.19 (0.90, 1.57)	1.14 (0.86, 1.51)	.382
Model 3, OR (95% CI)	1 (ref.)	1.09 (0.83, 1.45)	1.17 (0.88, 1.56)	1.13 (0.85, 1.50)	.425
From the two lowest categories to the highest^γ^					
Model 1, OR (95% CI)	1 (ref.)	1.39 (0.88, 2.18)	1.47 (0.93, 2.31)	1.86 (1.20, 2.87)	.006
Model 2, OR (95% CI)	1 (ref.)	1.37 (0.86, 2.19)	1.54 (0.96, 2.47)	1.96 (1.24, 3.07)	.003
Model 3, OR (95% CI)	1 (ref.)	1.50 (0.93, 2.42)	1.56 (0.96, 2.52)	2.00 (1.26, 3.16)	.005

The generalized ordered logistic model (gologit/partial proportional odds model) allows for the no equality of slopes among categories, being less restrictive and more flexible than the ordinal ordered logistic model (parallel-lines model). *OR* odds ratio, *CI* confidence interval

Model 1: logistic regression model adjusted for age

Model 2: as in model 1 and additionally adjusted for marital status, education, smoking, physical activity, sleep duration during weekdays and during the weekend, alcohol consumption, total fiber intake, cholesterol intake, and total energy intake

Model 3: as in model 2 and additionally adjusted for cardiovascular risk factors: total serum cholesterol, HDL serum cholesterol, systolic and diastolic blood pressure, body mass index, and diabetes

*Energy adjusted by the residual method

^†^Lowest category: CACS of 0; the two highest categories: CACS > 0 and < 100 together with CACS ≥ 100

^γ^Two lowest categories: CACS of 0 together with CACS > 0 and < 100; highest category: CACS ≥ 100

**Fig. 2:**
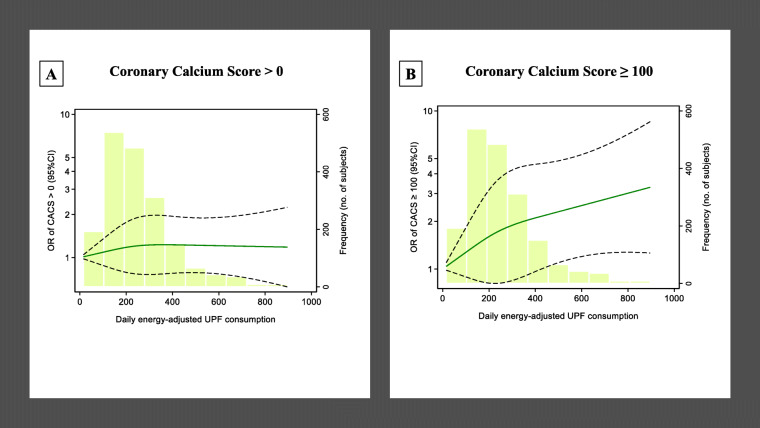
Restricted cubic splines for the association of Coronary Artery Calcium (CACS) and ultra-processed food (UPF) consumption, in the AWHS cohort study (*N* = 1876). The standard binary logistic regression was used to perform restricted cubic splines with 3 knots of the distribution (at the 10th, 50th, and 90th percentiles of the distribution). Participants with an exposure above the 99th percentile were not included. Dashed lines represent 95% CIs. The histograms show the distributions of energy-adjusted ultra-processed food consumption. **a** The odds of CACS > 0 (*p* value of the Wald test for non-linearity is 0.59) and **b** the odds of CACS > 100 (*p* value of the Wald test for non-linearity is 0.029) as ultra-processed food consumption increases. Models adjusted for age, marital status, education, smoking, physical activity, sleep duration during weekdays and during the weekend, alcohol consumption, total fiber intake, cholesterol intake, total energy intake, and cardiovascular risk factors: total serum cholesterol, HDL serum cholesterol, systolic and diastolic blood pressure, body mass index, and diabetes. AWHS, Aragon Workers’ Health Study; CACS, Coronary Agatston Calcium Score; UPF, ultra-processed food; OR, odds ratio; CI, confidence interval

Similar results were observed when performing the standard binary logistic regression to estimate OR for CACS > 0 (with CACS of 0 as reference) and for CACS ≥ 100 (with CACS < 100 as reference) (Additional File 1:Table S2). Thus, a clear dose-response relationship between UPF consumption and coronary artery calcium was only appreciated for CACS ≥ 100 compared to those with CACS < 100 (Fig. 2).

No interactions were detected between UPF consumption and age (*p* = 0.68), BMI (*p* = 0.25), smoking status (*p* = 0.15), alcohol consumption (*p* = 0.31), and physical activity (*p* = 0.61).

## Discussion

This is the first epidemiological study assessing the association between UPF consumption and early subclinical atherosclerosis. In this sample of middle-aged male workers, those with the highest consumption of UPF (on average approximately 500 g/day) compared with those with the lowest consumption (on average approximately 100 g/day) have a 2-fold increased risk of having a subclinical coronary atherosclerosis outlined by a CACS ≥ 100. This association was independent of the main cardiometabolic risk factors (cholesterol level, blood pressure, BMI, and diabetes), as well as dietary and lifestyle factors. Results were robust and remained similar using different methodological approaches. When we categorized the coronary calcium as CACS ≤ 0 vs. CACS > 0, we classified as having a “positive outcome”/“positive in atherosclerosis” a very heterogeneous group of participants with mixing characteristics, including those with CACS very close to 0 and those with mild, moderate, and even severe subclinical coronary atherosclerosis. This lack of specification and the fact that subjects with very small and incipient lesions (and in some cases with the coronary calcium almost expected by age) are classified as “positive outcome”/“positive in atherosclerosis” may be the reason for not having found an association when comparing CACS > 0 vs. CACS ≤ 0. In contrast, in the second categorization (CACS < 100 vs. CACS ≥ 100), by establishing the cutoff point at a CACS equal to 100, we classified as “positive outcome”/“positive in atherosclerosis” those with a well-defined and more established disease.

Scientific evidence has placed fresh fruit, vegetables, whole grains, nuts, legumes, and olive oil—for their high content of fiber and micronutrients with antioxidant and anti-inflammatory properties—as the most protective foods for the cardiovascular system [42, 43]. Despite all this knowledge, during the last decade, there has been a worldwide rapid increase in the consumption of UPF, in which these healthy components are scarce, due to the prevailing fact that UPF is easily accessible, tasty, and cheap [44].

Of particular concern is that (i) UPF is highly energy dense and is usually consumed in large portion sizes; (ii) UPF contains excessive salt, saturated fat, and refined sugars, while lacking fiber and micronutrients such as vitamins and antioxidants; (iii) additionally, potentially harmful compounds may be added or generated during UPF processing (such as colorants, additives, acrylamide, trans-fatty acids (TFAs)). Several of these UPF characteristics are known risk factors for cardiometabolic conditions [15], as we explain next.

To begin with, dense food and sugar-sweetened beverages might delay the trigger of the internal satiety signal, leading to excessive caloric ingestion [45]. Excessive intake of energy, fat, and sugar contributes to weight gain and increases the risk of obesity [16], which is a major risk factor for CVD. However, the associations observed in this study between consumption of UPF and subclinical coronary atherosclerosis were statistically significant even after adjusting for BMI. Thus, BMI does not fully explain the association between UPF and subclinical atherosclerosis.

Most of the salt intake in high-income nations comes from UPF. Among them, bread and bakery products, cereals and grains, meat products, and dairy products are the most significant contributors to dietary salt [46]. The high salt content of these industrial products may also partly contribute to the appearance of hypertension [47], endothelial dysfunction [22, 48], and, as a consequence, also CVD [49, 50].

Moreover, for the industrial production of UPF, vegetable oils (the most used due to their low cost) are hydrogenated. If the hydrogenation is total, saturated fat is formed, but if the hydrogenation is partial, TFAs are also produced. These partially hydrogenated oils are consumed in margarine, fast food, and other UPF such as cakes, rolls, confectionery, biscuits, chocolate, potato chips, and crisps. A relationship between TFA intake and increased risk of CVD has been established [51, 52]. In a meta-analysis of prospective studies, a 2% increase in total daily energy intake from TFAs was associated with a 23% increased risk of CVD [53]. In addition, it has been observed that TFAs increase the incorporation of calcium to vascular endothelium cells [54]. Likewise, controlled dietary trials have shown that TFAs have markedly adverse effects on serum lipids [55], raising serum concentrations of LDL cholesterol [56], while also decreasing the serum concentration of HDL cholesterol [57]. TFAs have also shown to rise inflammation markers including C-reactive protein (CRP), interleukin-6 (IL-6), and tumor necrosis factor-alpha (TNF-α) [58]. All these factors contribute independently to the development of atherosclerosis [22]. It should be mentioned, however, that the food industry has made remarkable efforts to reduce TFAs these past few years. Consequently, their presence in current UPF may be small [59].

UPF is also much rich in saturated fatty acids (SFAs) than in monounsaturated fatty acids (MUFAs) or polyunsaturated fatty acids (PUFAs). Higher intake of SFAs from pastries and processed food has been associated with a higher risk of CVD [60]. Likewise, the isocaloric substitution of SFAs or TFAs with MUFAs or PUFAs has been consistently associated with a lower risk of CVD and death, both in cohort studies [61] and in randomized controlled trials [62] .

Refined sugars and sweeteners added in UPF have been described as implicated in the development of CVD [63, 64] and its risk factors, such as hypertension [65], diabetes [66], metabolic syndrome [67], and obesity [64].

Food additives in UPF are of special concern. Some of them, such as sulphites [12] and monosodium glutamate [11], have shown several adverse effects on cardiovascular health in experimental studies on cellular models and animals. Likewise, it has been found that long-term consumption of acesulfame K (non-caloric artificial sweetener) might accelerate atherosclerosis in cellular models [10]. However, among the additives, phosphates deserve special mention [68]. UPFs with high amounts of added phosphates are processed meat, ham, sausages, canned fish, baked goods, cola drinks, and other soft drinks. Phosphorus in inorganic phosphate coming from UPF is very effectively absorbed in the gastrointestinal tract (absorbed by approximately 90%) with respect to naturally occurring phosphorus in food (absorbed by 40–60%) [69]. Phosphate induces vascular calcification both in vitro and in vivo [13, 70]. The promoted process is not merely the passive precipitation of calcium by phosphate, but rather an active cellular process in which smooth-muscle cells in blood vessels are reprogrammed to become osteoblast-like cells [13]. This alteration also seems to occur in the human arteries [71, 72]. Moreover, it has been shown that increased phosphate intake leads to an endothelial-cell function impairment in the vascular system, in both animals and humans [73]. High to normal serum phosphate concentrations are associated with coronary calcification in young healthy men [74] and were found to be a predictor of cardiovascular events in the Framingham study [75].

Neo-formed substances which are produced through packaging, moisture removal, heat treatments, chilling and freezing, acidity control, reaction with chemical additives, and irradiation might also contribute to the harmful effect derived from UPF consumption. An example of a neo-formed substance from packaging is bisphenol A, which has been related to CVD [76]. Likewise, acrylamide, which is produced by heat treatments and found in fried potatoes, biscuits, bread, processed meat, and even coffee, has also been associated with CVD [77, 78].

Further research is needed to identify what specific processes, compounds, or UPF subtypes play an important role in this association found between UPF consumption and increased risk of early atherosclerosis, also taking into account the possibility of synergic effects among the abovementioned mechanisms. This knowledge will allow, in the short term, to recommend a reduction of the consumption of these products and, in the long term, to refine the processes of the food industry with the aim of providing a healthier offer.

Some limitations of our study must be recognized. First, the cross-sectional design of our study prevents us from establishing a causal link between consumption of UPF and subclinical coronary atherosclerosis. However, since calcium in the coronary artery is subclinical, reverse causation is highly unlikely. Also, although we adjust for a wide range of potential confounders, we cannot rule out residual confounding.

Although the FFQ provides an adequate assessment of an individual’s usual diet [35], because of its self-reported nature and the potential recall bias, inaccuracies in the exposure assessment cannot be ruled out. Likewise, the FFQ was not designed specifically to collect data on UPF following the methodology of the NOVA classification, which could lead to misclassification. Nonetheless, the applied methodology is the most frequently used classification of UPF in epidemiological studies. Despite the NOVA classification being reproducible and easily incorporated into messages and its consequent utility for public health, it is not exempted from controversies [79, 80].

We should note that the CACS fails to capture information about the regional distribution of calcification within the coronary tree and does not incorporate information on the number or size of calcified coronary lesions. The limited external validity of our findings should also be mentioned, as the cohort was not representative of the general population.

This study notably presents important strengths, such as its novelty and the quality of the methodology used to collect clinical data and to quantify coronary calcium, which is a measurement with strong published support of its value for clinical risk prediction. Also, the detailed data collection for confounders, including accurate measurements of blood pressure and serum lipids, helps reduce confounding. Finally, another asset is the consistency of the findings after using different statistical approaches to assess the association between UPF and subclinical atherosclerosis.

## Conclusion

In conclusion, in this study of asymptomatic Spanish working men, we found that those consuming the highest amount of UPF had twice as much probability of having subclinical coronary atherosclerosis, regardless of blood lipids, hypertension, BMI, and other cardiovascular risk factors.

## Supplementary information

**Additional file 1: Table S1** Food-items included as UPF; **Table S2** UPF-CACS association.

## Data Availability

The datasets used and/or analyzed during the current study are available from the corresponding author on reasonable request.

## Abbreviations

AWHS: Aragon Workers’ Health Study
BMI: Body mass index
CACS: Agatston's Coronary Artery Calcium Score (CACS)
CI: Confidence interval
CVD: Cardiovascular disease
OR: Odds ratio
UPF: Ultra-processed food

## Supplementary information

**Table S1** displays the food-items in the AWHS food frequency questionnaire classified as ultra-processed foods according to degree of processing (NOVA group 4). **Table S2** Association between Agatston's Coronary Artery Calcium Score and quartiles of UPF consumption by using the standard binary logistic regression. The =R(95% CI) for CACS > 0 (compared with CACS score ≤ 0) and for CACS score ≥ 100 (compared to CACS score < 100) are displayed. **Supplementary information** accompanies this paper at 10.1186/s12916-020-01678-8.
