# Supplementary material for: High consumption of ultra-processed food may double the risk of subclinical coronary atherosclerosis: the Aragon Workers’ Health Study (AWHS)
Source: BMC Med. 2020 Aug 13;18:235. doi: 10.1186/s12916-020-01678-8 (PMC7425006; doi:10.1186/s12916-020-01678-8)
Supplement: Supplementary file 1 — Additional file 1: Table S1 Food-items included as UPF; Table S2 UPF-CACS association. [file 12916_2020_1678_MOESM1_ESM.pdf]

Additional File 1

High Consumption of Ultra-Processed Food Doubles the Risk of Subclinical Coronary Atherosclerosis: The Aragon Workers Health Study (AWHS)

Henry Montero Salazar[1], Carolina Donat-Vargas[1,2], Belén Moreno-Franco[3,4] Helena Sandoval-Insausti[1,6], Fernando Civeira[3,4,5], Martín Laclaustra[3,4,5], Pilar Guallar-Castillón[1,7,8,9]

1. Department of Preventive Medicine and Public Health. School of Medicine. Universidad Autónoma de Madrid. CEI UAM+CSIC, Madrid. Spain.
2. Unit of Nutritional and Cardiovascular Epidemiology, Environmental Medicine Institute (IMM), Karolinska Institutet. Estocolm. Sweden.
3. IIS Aragón, Hospital Universitario Miguel Servet, Universidad de Zaragoza, Spain
4. CIBERCV Instituto de Salud Carlos III. Madrid, Spain
5. Agencia Aragonesa para la Investigación y el Desarrollo (ARAID), Zaragoza, Spain
6. Department of Nutrition, Harvard T.H. Chan School of Public Health, Boston, MA, USA.
7. CIBERESP (CIBER of Epidemiology and Public Health) Instituto de Salud Carlos III. Madrid, Spain
8. Instituto de Investigación IdiPaz, Madrid. Spain
9. IMDEA-Food Institute. Madrid, Spain

Additional File 1: Table S1.

Food-items in the AWHS food frequency questionnaire classified as ultra-processed foods according to degree of processing (NOVA group 4)

Petit Suisse; custard; flan; pudding; ice cream; ham; lard and derivatives, processed meat (chorizo, salami, mortadella, sausage, hamburger, morcilla (blood pudding); pate; snack; foie-grass; spicy sausage/meatballs; commercial potato chips; breakfast cereals; pizza, including pre-prepared pies; margarine; cookies; whole meal cookies; chocolate cookies; muffins; jam; doughnuts; croissant or other non-handmade pastries; cakes; churros; chocolates and candies; nougat; marzipan; carbonated drinks; artificially sugared beverages; fruit drinks; milkshakes; instant soups and creams; croquettes; mayonnaise; mustard, and alcoholic drinks produced by fermentation followed by distillation such as whisky, gin, and rum

Additional File 1: Table S2.
Association of Coronary Artery Calcium and quartiles of ultra-processed food consumption , in the AWHS cohort study (N=1,876)

	CACS > 0					CACS ≥ 100				
Cases/N	747/1,876					191/1,876				
**Energy-adjusted UPF consumption (g/day)**	**Q1**	**Q2**	**Q3**	**Q4**	***P* trend**	**Q1**	**Q2**	**Q3**	**Q4**	***P* trend**
Model 1, OR (95%CI)	1 (ref.)	1.10 (0.84, 1.44)	1.18 (0.90, 1.54)	1.15 (0.88, 1.51)	.317	1 (ref.)	1.33 (0.84, 2.09)	1.41 (0.90, 2.23)	1.93 (1.24, 2.99)	.003
Model 2, OR (95%CI)	1 (ref.)	1.10 (0.83, 1.45)	1.18 (0.89, 1.56)	1.14 (0.86, 1.51)	.363	1 (ref.)	1.31 (0.82, 2.10)	1.45 (0.91, 2.32)	1.93 (1.22, 3.03)	.004
Model 3, OR (95%CI)	1 (ref.)	1.11 (0.84, 1.47)	1.18 (0.89, 1.56)	1.15 (0.86, 1.53)	.368	1 (ref.)	1.38 (0.86, 2.23)	1.43 (0.88, 2.30)	1.98 (1.25, 3.15)	.004

Logistic regression was used to estimate the Odds Ratios (95% CI) for CACS>0 (compared with CAC score ≤ 0) and for CAC score ≥100 (compared to CAC score <100).
Model 1: Logistic regression model adjusted for age
Model 2: as in Model 1 and marital status, education, smoking, physical activity, sleep duration during weekdays and during weekend, alcohol consumption, total fiber intake, cholesterol intake, and total energy intake.
Model 3: as in Model 2 and additionally adjusted for total serum cholesterol, HDL serum cholesterol, systolic and diastolic blood pressure, body mass index, and diabetes.
AWHS: Aragon Workers' Health Study, **CACS**: Coronary Calcium Score; **UPF**: Ultra-processed Food; **OR**: Odds Ratio; **CI**: Confidence Interval.
